# Robust Point Cloud Registration via Rotation-Equivariant Geometric Encoding and State Space Models

**DOI:** 10.3390/jimaging12050214

**Published:** 2026-05-18

**Authors:** Junjie Li, Jiajun Liu, Anqi Chen, Huifang Shen, Jianya Yuan

**Affiliations:** 1 College of Mechanical and Electrical Engineering, Fujian Agriculture and Forestry University, Fuzhou 350108, China; 52312047019@fafu.edu.cn (J.L.); 52412047005@fafu.edu.cn (J.L.); 52412047001@fafu.edu.cn (A.C.); 2 Quanzhou Institute of Equipment Manufacturing, Haixi Institutes, Chinese Academy of Sciences, Quanzhou 362216, China; yuanjianya@fjirsm.ac.cn; 3 Fujian Institute of Research on the Structure of Matter, Chinese Academy of Sciences, Fuzhou 350025, China; 4 Fujian College, University of Chinese Academy of Sciences, Fuzhou 350025, China

**Keywords:** point cloud registration, rotation equivariance, state space model, geometric encoding, three-dimensional computer vision

## Abstract

Point cloud registration in environments lacking rich textures or containing repetitive structures remains highly susceptible to misalignments. The core challenge lies in balancing the demand for extracting highly distinctive local features with the computational cost of global context modeling. In this paper, we propose a robust registration framework that efficiently combines rotation-equivariant geometric representations with state space models of linear complexity to mitigate feature ambiguity and mismatch. First, a multivariate geometric encoding mechanism is embedded within convolutional layers, enhancing local feature distinctiveness under strict rotation equivariance by explicitly leveraging surface properties. Second, to efficiently establish long-range spatial dependencies, we replace standard dense attention with a hybrid geometry-state aggregation module. This module integrates local geometric self-attention with the Mamba architecture, strengthening focus on overlapping regions without the quadratic computational burden. Finally, we optimize the generated correspondences through a physically consistent hypothesis generator to compute reliable rigid transformation results. On standard benchmarks, our framework demonstrates exceptional robustness to ambiguous matches, achieving a 96.3% registration recall on the 3DMatch dataset and outstanding accuracy on the KITTI dataset.

## 1. Introduction

Three-dimensional point cloud registration is a pivotal task in computer vision, aiming to estimate a rigid transformation matrix that aligns two partially overlapping point clouds captured from different viewpoints into a unified coordinate system. This technology has found widespread applications in critical fields such as forestry monitoring [1], 3D reconstruction [2], medical imaging analysis [3], and environmental perception for autonomous driving [4]. Recently, driven by the powerful representation capabilities of deep neural networks, learning-based registration paradigms have achieved remarkable progress, gradually replacing traditional optimization-based methods. Intuitively, extracting robust geometric features from unstructured points is fundamental to achieving accurate registration.

To extract more discriminative representations, deep learning-based registration methods have been extensively explored [5,6,7]. Fully convolutional geometric feature networks [8] significantly improve inference speed by capturing spatial context through compact descriptors. RoCNet++ [9] constructs geometric feature descriptors by performing a nearest neighbor search for each point to obtain all surrounding triangles, and subsequently calculating the angles and distances. Han et al. [10] incorpore at point cloud color information into their model as a guide for descriptor construction and eliminate misalignments through color consistency. Accurately capturing underlying topological structures during local feature extraction relies heavily on the definition of the neighborhood space. Existing studies validate that dynamic or variable neighborhood selection significantly enhances feature extraction quality [11]. To address the fundamental challenges of non-uniform density and complex topologies in 3D point clouds, prior deep learning works have actively explored adaptive modeling strategies. For instance, DGCNN [12] dynamically updates graph connections in the feature space rather than relying solely on initial spatial proximity. Similarly, KPConv [13] introduces deformable convolutions that learn local shifts to adapt kernel points specifically to the local geometry. To achieve feature adaptability without altering the spatial graph, PAConv [14] dynamically assembles convolution kernels using implicit positional mapping learned from relative point positions. While these adaptive mechanisms provide remarkable flexibility, they are not inherently robust to arbitrary 3D rotations.

Recently, various rotation-equivariant networks have been developed to fundamentally address rotation sensitivity. This class of networks employs carefully designed rotation-equivariant mathematical operations, such as introducing group convolutions [15] or specific isometry designs [16], enabling the network’s output features to undergo synchronous, predictable rotations alongside the input point cloud, partially resolving the matching ambiguity caused by pose variations. Nevertheless, existing rotation-equivariant models still struggle to balance local geometric details and global contextual information during feature extraction. This challenge is particularly pronounced in scenes containing repetitive structures or weak texture regions. In such environments, the distinctiveness of the extracted local feature descriptors is severely degraded, inevitably leading to ambiguous correspondences and overall registration failure.

To enhance global perception capabilities, the Transformer [17] architecture has been widely adopted in point cloud registration [18], leveraging self-attention mechanisms to capture long-range dependencies. Despite its impressive performance, the quadratic computational complexity $O(N^{2})$ of attention mechanisms results in substantial memory overhead and inference latency. Recently, State Space Models (SSMs), particularly the Mamba architecture [19], have provided new insights for point cloud analysis due to their linear complexity and powerful sequence modeling capabilities [20]. However, existing Mamba adaptation schemes in the point cloud domain are primarily designed for classification tasks, and their serialization strategies may disrupt the topological consistency of geometric structures, which is essential for registration tasks. Therefore, there is a critical need to deeply integrate explicit geometric structures with efficient linear state space models to achieve high-accuracy, efficient, and robust registration.

In this paper, we propose a novel deep learning framework that achieves highly robust 3D point cloud registration. It aims to enable the network to extract more discriminative features through explicit physical geometric modeling, and effectively combines the geometric anchoring capability of Transformers with the global modeling capability of a state space model to achieve robust point correspondence matching.

To realize this, we introduce three key innovations. First, to tackle the issue of insufficient local geometric descriptive power, we integrate a Multivariate Geometric Combination Encoding (MGCE) mechanism into the rotation-equivariant convolution, termed MG-Conv. Unlike approaches that rely on complex aggregation modules, which may disrupt equivariance, MG-Conv explicitly incorporates multivariate geometric features into the generation process of dynamic kernel weights via scalar embedding. This design enables the convolution to adaptively perceive surface variations, significantly enhancing feature distinctiveness while strictly preserving rotation-equivariance. Second, we propose a Hybrid Geometry-State Aggregation Module (HGSAM). This module replaces fully connected self-attention with local self-attention to capture geometric structure, while leveraging Mamba architecture with linear complexity to aggregate global topological information. This decoupled design ensures efficient complementarity between precise local geometry and broad global semantics. Furthermore, we employ a Physically Consistent Robust Hypothesis Proposer (PCRH-P) to mitigate the robustness issues in the backend pose estimation, such as spatially clustered hypotheses and convergence oscillations. We introduce a spatial diversity sampling strategy to mitigate clustering, coupled with early pruning of invalid correspondences based on rotation-invariant feature consistency norms, and adopt a soft-weighted refinement scheme to achieve a smoother and more accurate convergence process in the final alignment stage. The main contributions of this paper are summarized as follows:

- We propose MG-Conv, a rotation-equivariant convolution that explicitly computes and aggregates intrinsic geometric attributes. It significantly enriches the discriminative power of local features without compromising the network’s global equivariance.
- We construct HGSAM, a hybrid module that decouples geometric modeling from context aggregation. By combining the precision of Transformers with the linear efficiency of Mamba, this module enables the model to focus more on overlapping regions while maintaining low computational complexity.
- We develop PCRH-P, incorporating spatial diversity sampling, consistency pruning, and soft-weighted refinement to substantially improve the reliability and stability of pose estimation.
- Extensive experiments on indoor and outdoor datasets demonstrate that our method outperforms existing approaches.

## 2. Related Works

The evolution of 3D point cloud registration has transitioned from classical geometry-based optimization to sophisticated deep learning paradigms that prioritize robustness and efficiency. At its core, the registration problem is fundamentally centered around establishing reliable point-wise or patch-wise correspondences between disparate scans to recover the optimal rigid transformation. Traditional algorithms primarily rely on hand-crafted geometric descriptors and iterative optimization. To robustly estimate the final rigid transformation matrix from initial matches highly contaminated by outliers, the Random Sample Consensus (RANSAC) [21] and its extended algorithms [22] have always been indispensable core mechanism in 3D vision. However, these methods often exhibit limited generalization when encountering repetitive structures or low-overlap conditions. In such challenging environments, the scarcity of highly discriminative geometric cues leads to a proliferation of incorrect matches. Consequently, the registration process becomes highly susceptible to outliers and frequently suffers from entrapment in local optima. To address these challenges, the research focus has increasingly shifted toward learning-based methodologies that can autonomously encode complex structural patterns and capture global spatial dependencies. This paradigm shift has motivated the exploration of more expressive feature extractors and efficient aggregation architectures.

### 2.1. Feature Extraction

Early learning-based methods [23,24,25] emerge as efficient alternatives to traditional optimization-based approaches, offering significant speed improvements. However, these early works primarily relied on global feature aggregations, making them inherently fragile when encountering arbitrary 3D rotations in unconstrained scenarios. To address this, methods based on local reference frames, such as RIGA [26], are proposed to canonically align inputs into a unified coordinate system, achieving a degree of rotation invariance. Subsequently, to further mitigate sensitivity to pose variations, rotation-equivariant networks were introduced to provide a stronger model inductive bias. BUFFER [27] pioneers the use of Vector Neurons (VNs) [28] for point-level equivariance combined with a lightweight Mini-SpinNet [29] for patch features. YOHO [16] and RoReg [15] leverage the closure property of groups to extract point features under 60 different poses, yielding rotation-invariant and rotation-equivariant group features, enabling the network to estimate transformation matrices from a single correspondence hypothesis. PARE-Net [30] incorporates a lightweight position-aware convolution to extract rotation-invariant spatial information and rotation-equivariant group features of local structures. Zhang et al. [31] utilize 4D Point Pair Features (PPF) to extract rotation-invariant local descriptors, which serve as a guide for the global feature matching process. While these methods achieve rigorous geometric robustness, they primarily rely on local receptive fields.

### 2.2. Transformer on Registration

To circumvent the receptive field limitations of local descriptors, Transformer-based architectures [18,32] have been widely adopted to capture long-range dependencies. Early works like Predator [33] and CoFiNet [34] leverage attention mechanisms to implicitly model overlap and establish correspondences. Moving towards end-to-end paradigms, REGTR [35] replaces explicit RANSAC-based matching with a transformer network that directly predicts point correspondences and their probabilities of lying in overlapping regions. However, pure attention often neglects explicit geometric topology. Consequently, recent research focuses on injecting geometric priors into Transformers. RoITR [36] integrates PPF into the attention layer. Most notably, GeoTransformer [37] proposes a geometric self-attention module that embeds rotation-invariant structures, achieving state-of-the-art robustness. Subsequent methods have further explored embedding coordinates [38,39] or semantic cues [40] to enrich feature representation. To address the computational complexity of Transformers in large-scale scenarios, RegFormer [41] proposes an efficient projection-aware Transformer network by extracting features from projected views.

### 2.3. State Space Models in 3D Vision

Recently, Mamba has emerged as a compelling alternative to Transformers, characterized by its linear complexity in sequence modeling [42]. Despite its success in 2D vision [43], the extension of Mamba to 3D point cloud analysis is still in its early stages. Pioneering works like PointMamba [20] and PoinTramba [44] have adapted SSMs for classification and segmentation, typically introducing specific reordering strategies to mitigate point disorder. However, such strategies are ill-suited for registration tasks. The serialization process in these methods inevitably shatters the spatial adjacency of local neighborhoods, leading to a loss of fine-grained geometric topology. Therefore, effectively leveraging Mamba’s global efficiency without compromising the local geometric fidelity required for dense matching remains an open research problem.

## 3. Methods

Given a source point cloud $P={\left\{p_{i}\in R^{3}\right\}}_{i=1}^{N}$ and a target point cloud $Q={\left\{q_{j}\in R^{3}\right\}}_{j=1}^{M}$ scanned from partially overlapping scenes, the goal of rigid point cloud registration is to recover the optimal rigid transformation T=[R,t], composed of a rotation R∈SO(3) and a translation $t\in R^{3}$, that aligns P to Q by minimizing the alignment error of corresponding points.

**Figure 1:**
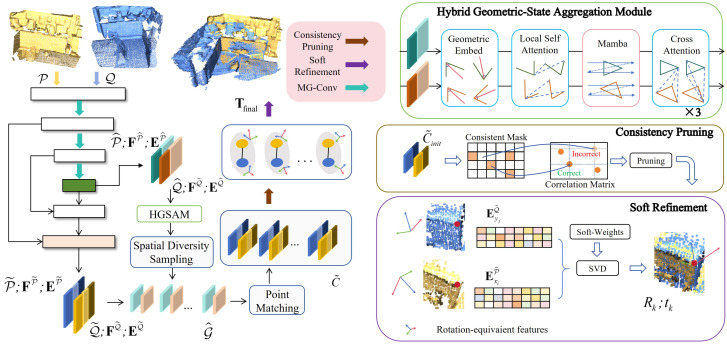
The overall architecture of the proposed network. The pipeline consists of three main stages: (1) Input point clouds P and Q are processed by a hierarchical encoder using MG-Conv to generate superpoints $\hat{P}$ and $\hat{Q}$. Specifically, the network explicitly extracts both rotation-invariant features ($F^{\hat{P}},F^{\hat{Q}}$) and rotation-equivariant features ($E^{\hat{P}},E^{\hat{Q}}$) for the superpoints, alongside the corresponding rotation-invariant ($F^{\tilde{P}},F^{\tilde{Q}}$) and rotation-equivariant features ($E^{\tilde{P}},E^{\tilde{Q}}$) for the respective dense point sets. (2) The coarse-level features are fed into HGSAM for feature interaction, after which superpoint matches $\hat{G}$ are computed to constrain the search space for fine-grained dense point matching $\tilde{C}$. (3) The PCRH-P module directly leverages their point-wise rotation-equivariant features ($E_{x_{i}}^{\tilde{P}},E_{y_{j}}^{\tilde{Q}}$) from ${\tilde{C}}_{\mathrm{init}}$ to compute a set of transformation hypotheses $\left\{\left[R_{k},t_{k}\right]\right\}$ via Singular Value Decomposition (SVD) [46]. The final transformation $T_{\mathrm{final}}$ is computed through soft-weighted refinement.

**Figure 2:**
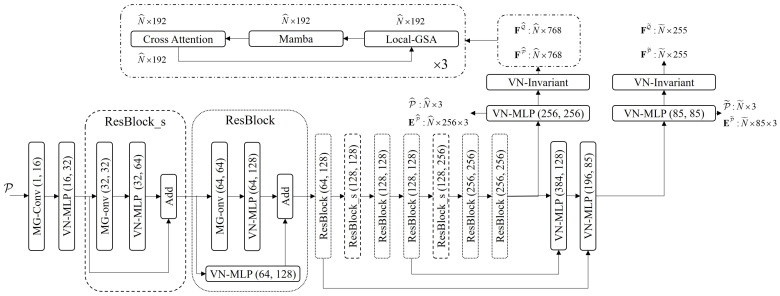
The pipeline features a hierarchical pyramid backbone and a hybrid interaction stage. To maintain rotational equivariance while deepening the network, we design two types of residual blocks: the standard Residual Block (ResBlock) and the Strided Residual Block (ResBlock_s). Both modules are composed of the MG-Conv operator and VN-MLP to extract rotation-equivariant features, while rotation-invariant features are extracted through the VN-Invariant block, with specific input and output channels annotated for each stage. The feature interaction is performed by a three-layer HGSAM module, where the tensor dimensions are maintained at $\hat{N}\times 192$ during the Local-GSA, Mamba, and Cross-Attention operations.

The overall architecture of the proposed framework is illustrated in Figure 1. We construct a hierarchical pyramid backbone network based on MG-Conv to extract multi-level, geometrically enriched features. To effectively reduce computational complexity and enlarge the receptive field of the network while preserving key geometric structures, we apply downsampling strategies to the input point clouds, which is a fundamental operation in prevalent hierarchical feature learning frameworks for point clouds [45]. Distinct from random sampling, which often leads to structural sparsity and feature instability, we explicitly employ a voxel grid algorithm for a four-layer sequential downsampling process to ensure a spatially uniform representation. This uniformity provides the necessary physical basis for MG-Conv to capture precise local topologies. Within this architecture, MG-Conv modules extract rotation-equivariant features E and rotation-invariant features F at each level. We also leverage a feature propagation mechanism with nearest-neighbor upsampling indices to map these features back to the dense point sets, denoted as $\tilde{P}$ and $\tilde{Q}$. At the coarsest level, superpoints and their rotation-invariant features are fed into the HGSAM module, iteratively employing local geometric self-attention, global Mamba and cross-attention to focus on overlapping regions between two point cloud frames. As illustrated in Figure 2, we provide a detailed visualization of the MG-Conv backbone and HGSAM architecture of our method.

### 3.1. Multivariate Geometric Feature Extraction

To enhance the distinctiveness of local features while maintaining rotation equivariance, we introduce a dual-stream extraction strategy. This approach explicitly models surface properties to guide the learning of local descriptors. The proposed extraction framework is detailed in two stages: the explicit encoding of physical invariants via the MGCE module, followed by its seamless integration into the MG-Conv layers to maintain strict rotation equivariance while enhancing descriptive power.

#### 3.1.1. Multivariate Geometric Combination Encoder

**Figure 3:**
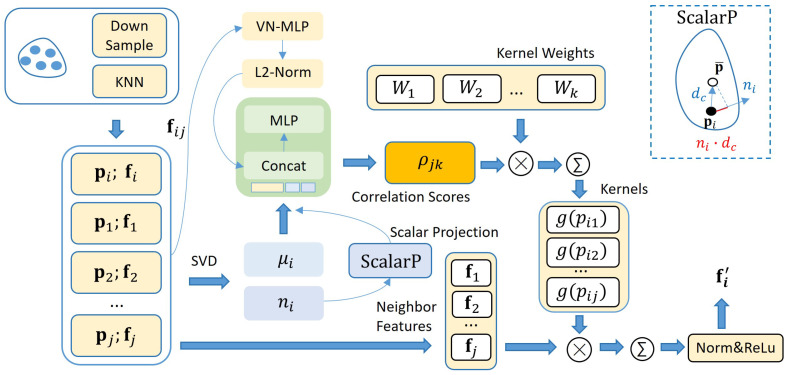
The illustration of MG-Conv module. For a given center point $p_{i}$ and its local neighborhood $N_{i}$, the module explicitly captures spatial neighborhood features $f_{j}$, surface normals $n_{i}$ and curvature $\mu_{i}$ from local point cloud patches. The extracted geometric attributes are transformed into a rotation-invariant scalar descriptor via relative projection. It is subsequently injected into the dynamic kernel generation process, ultimately yielding distinctive rotation-equivariant features $f_{i}^{\prime }$. The red line segment represents the projection of the centroid offset vector $d_{c}$ in the normal direction $n_{i}$.

Existing rotation-equivariant networks primarily rely on implicit feature learning, often failing to capture fine-grained topological details. To bridge this gap, we propose the MGCE mechanism to incorporate explicit geometric constraints into feature extraction. As illustrated in Figure 3, MGCE employs eigendecomposition to explicitly capture the physical invariants of local surfaces. These attributes are encoded as rotation-invariant scalars to condition the dynamic kernel generation of MG-Conv, thereby augmenting the network’s geometric perception capabilities beyond purely implicit feature-based approaches.

Specifically, for a center point $p_{i}$ and its K-nearest neighbor set $N_{i}={\left\{p_{j}\right\}}_{j=1}^{K}$, we first compute the local centroid $\bar{p}=\frac{1}{K}\sum p_{j}$. Then, the local covariance matrix is formulated as

(1) $$M_{i}=\frac{1}{K}\sum_{p_{j}\in N_{i}}\left(p_{j}-\bar{p}\right){\left(p_{j}-\bar{p}\right)}^{⊤}.$$

Subsequently, because the input point cloud exists in 3D Euclidean space, the local covariance matrix $M_{i}$ is strictly a 3×3 matrix. Therefore, performing eigendecomposition on it yields exactly three eigenvalues $\lambda_{1}\geq \lambda_{2}\geq \lambda_{3}$ and their corresponding eigenvectors $u_{1},u_{2},u_{3}$. Here, $u_{1}$ and $u_{2}$ correspond to the largest and second-largest eigenvalues, which capture the directions of maximum spatial variance. Because the local points primarily spread along the actual surface geometry, these two principal directions naturally span the local tangent plane of the surface. Consequently, the third eigenvector $u_{3}$ corresponding to the smallest variance is strictly orthogonal to this tangent plane, making it the mathematically optimal and most compact representation of the surface normal $n_{i}$. Furthermore, we calculate the curvature intensity $\mu_{i}$ from the normalized eigenvalues using the standard formula $\mu_{i}=\frac{\lambda_{3}}{\lambda_{1}+\lambda_{2}+\lambda_{3}+u}$, where $u=10^{-7}$ is a small stabilizing constant to handle degenerate planar cases to quantify the extent to which a local neighborhood deviates from its tangent plane. To strictly preserve rotation equivariance and resolve the inherent sign ambiguity of PCA-derived normals between $n_{i}$ and $-n_{i}$, we avoid directly utilizing the rotation-variant normal vector $n_{i}$. Instead, we compute the absolute dot product between $n_{i}$ and the local centroid offset vector $d_{c}=\frac{1}{K}\sum_{p_{j}\in N_{i}}\left(p_{j}-p_{i}\right)$ to capture the morphological characteristics of the local surface. Geometrically, when the point cloud undergoes an arbitrary rotation R∈SO(3), both $n_{i}$ and $d_{c}$ rotate synchronously (i.e., $n_{i}^{\prime }=Rn_{i}$ and $d_{c}^{\prime }=Rd_{c}$). Due to the preservation of the inner product under orthogonal transformations, the absolute result $|n_{i}^{\prime }\cdot d_{c}^{\prime }\left|=\right|n_{i}\cdot d_{c}|$ remains constant, thereby projecting the rotation-variant vectors into a rotation-invariant scalar space. Finally, this scalar projection is concatenated with the curvature value to form the explicit geometric descriptor $S_{\mathrm{geo}}$:

(2) $$S_{\mathrm{geo}}=\mathrm{Concat}[\mu_{i},\;|n_{i}\cdot d_{c}\left|\right],$$

where Concat[·] denotes the channel-wise feature concatenation operation. Diagnostically, within $S_{\mathrm{geo}}$, the curvature $\mu_{i}$ acts as a topological sharpness indicator isolating corners and edges, while $|n_{i}\cdot d_{c}|$ encodes the relative spatial variation of the local patch. Since a smaller *K* introduces severe feature instability due to sensor noise, whereas a larger *K* over-smooths distinctive high-frequency geometric structures, we choose K=35 to provide an optimal balance.

#### 3.1.2. Multivariate Geometry-Guided Rotation-Equivariant Convolution

Based on explicit physical priors extracted via MGCE, we propose MG-Conv to inject inductive biases into the feature aggregation process. While MG-Conv adopts the dynamic kernel generation paradigm of PARE-Conv [30], it introduces fundamental improvements to the mechanism. Unlike PARE-Conv, which solely leverages implicit VN-MLP [28] to learn positional features for generating convolutional kernel weights, MG-Conv incorporates an explicit-implicit dual-stream fusion mechanism. This mechanism employs a dynamic kernel generation scheme in which kernel weight generation is not only determined by implicitly learned features but is also strictly conditioned by explicit physical geometry.

Specifically, we similarly concatenate the relative coordinate vector $p_{ij}=p_{j}-p_{i}$, local centroid offset vector $d_{c}$ and their cross product to construct a rotation-equivariant spatial feature $f_{ij}$. Then, we extract positional information through a standard VN-MLP to obtain the implicit features. To inject physical constraints, we perform channel-wise concatenation between the $L_{2}$-normalized implicit features and the explicit scalar output from the MGCE to construct a hybrid geometric descriptor. Subsequently, the hybrid descriptor is fed into a lightweight correlation network ϕ. The network ϕ consists of an MLP block and a softmax layer, which predicts the contribution weights $\rho_{jk}$, corresponding to the *k*-th kernel weight for the *j*-th neighbor:

(3) $$\rho_{jk}=\phi \left(\mathrm{Concat}\left[{∥\mathrm{VN}(f_{ij})∥}_{2},S_{\mathrm{geo}}\right]\right).$$

The network employs geometry-enhanced correlation weights to perform weighted aggregation and dynamic assembly within the rotation-invariant feature space, thereby achieving the process of injecting explicit geometric constraints into the generation of convolution kernels $W_{k}$. Crucially, since the correlation weights $\rho_{jk}$ we construct are rotation-invariant scalars, according to vector neuron theory, $\rho_{jk}W_{k}$ will not violate the network’s overall rotation equivariance, i.e., $f\left(R\cdot \rho_{jk}W_{k}X\right)=R\cdot f\left(\rho_{jk}W_{k}X\right)$, where X represents the input feature vectors or coordinates fed into the convolutional layer and f(·) denotes the feature mapping function of the convolutional layer. Ultimately, for each center point $p_{i}$ and its *j*-th neighboring point feature $f_{j}$ within the local region, the core operation of MG-Conv can be formulated as

(4) $$\left(F*g\right)\left(p_{i}\right)=\sum_{p_{j}\in N_{i}}\left(\sum_{k}\rho_{jk}W_{k}\right)\;f_{j},$$

Here, the left side of the equation denotes the continuous convolution operation evaluated specifically at the center point $p_{i}$. F represents the continuous input feature field across the entire point cloud, while *g* denotes the continuous convolution kernel function. The asterisk ∗ denotes the continuous convolution operation between them.

Compared to existing rotation-equivariant convolutions, MG-Conv establishes a synergy between explicit physical priors and implicit data-driven representations. Recent studies on point cloud surface representation, such as [47], have demonstrated that explicitly modeling local geometry is crucial for distinguishing complex topological structures, including planes, edges, and corners. Motivated by this insight, we transform explicit geometric features into rotation-invariant scalars via relative geometric projection to guide dynamic kernel weight generation, allowing the network to adaptively modulate the feature extraction process, significantly enhancing both structural perception and feature discriminability. Furthermore, this dynamic modulation strictly preserves mathematical SO(3) equivariance. As established in the Vector Neurons framework [28], scaling equivariant vectors by rotation-invariant scalars inherently maintains the overall group equivariance of the system. Subsequently, we also apply a VN-invariant layer to derive rotation-invariant scalar features from the equivariant vectors for the following feature interaction.

### 3.2. Hybrid Geometry-State Aggregation Module

**Figure 4:**
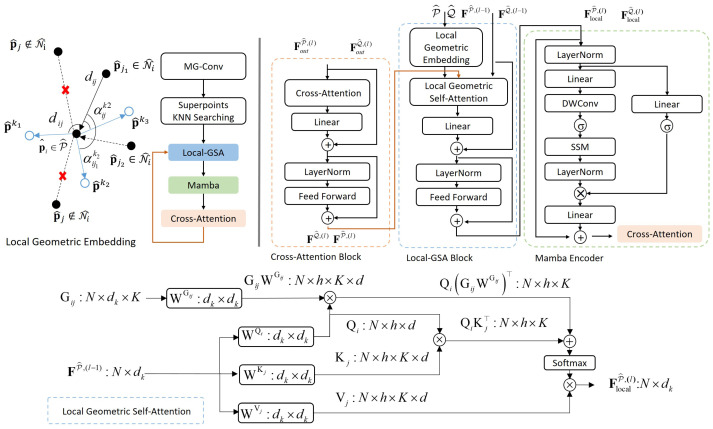
Architecture of the proposed HGSAM. The module integrates Local-GSA and Mamba Encoder to explicitly capture local geometry and global context, respectively, followed by a cross-attention module for inter-cloud feature interaction. In the Local Geometric Embedding diagram, solid black circles represent valid neighbor points and red crosses indicate excluded points outside the local neighborhood. Hollow blue circles denote the sampled superpoints.

We observe that geometric feature encoding exhibits inherent locality. Specifically, the geometric signature of a point is predominantly defined by its local neighborhood, while distant points contribute marginally to its structural description. Consequently, employing fully connected geometric attention incurs substantial computational redundancy. Conversely, completely abandoning long-range feature interaction hinders the model’s ability to capture global semantic context, which is crucial for disambiguating repetitive local geometries. To resolve this dilemma, we propose the HGSAM, a cascaded architecture that effectively decouples local geometric anchoring from global semantic diffusion. As illustrated in Figure 4, this module comprises three sequential subcomponents: a Local Geometric Self-Attention (Local-GSA) for robust local encoding, a global Mamba for efficient long-range modeling, and a cross-attention module for feature interaction.

Although MG-Conv extracts discriminative local geometric features, its limited receptive field makes it difficult to resolve matching ambiguities in scenes with repetitive structures. To address this issue, we formulate the HGSAM as a cascaded architecture with *L* stacked layers. Let $F^{\hat{P},\left(l-1\right)}$ and $F^{\hat{Q},\left(l-1\right)}$ denote the input superpoint features to the *l*-th iteration (l∈{1,…,L}). The initial inputs $F^{\hat{P},\left(0\right)}$ and $F^{\hat{Q},\left(0\right)}$ are initialized by the rotation-invariant features generated by MG-Conv. By providing the necessary global context, HGSAM effectively distinguishes structurally similar but spatially distant regions.

#### 3.2.1. Local Geometric Self-Attention

To capture fine-grained topological details while maintaining efficiency, we constrain the Local-GSA to operate exclusively within the $\hat{K}$-nearest neighbors. Given the input superpoint features $F^{\hat{P},\left(l-1\right)}={\left\{{\hat{f}}_{i}^{\left(l-1\right)}\right\}}_{i=1}^{\hat{N}}$ and their corresponding coordinates $\hat{P}={\left\{{\hat{p}}_{i}\right\}}_{i=1}^{\hat{N}}$, we first construct the local $\hat{K}$-neighborhood ${\hat{N}}_{i}$ for each superpoint ${\hat{p}}_{i}$. Following the formulation in GeoTransformer [37], we compute pairwise distances and triplet angles to construct the geometric structure embedding $G_{ij}$, ensuring invariance to rigid transformations. Distinct from the global approach, we compute this embedding strictly for local edges $j\in {\hat{N}}_{i}$, resulting in a sparse geometric graph:

(5) $$G_{ij}={\mathrm{MLP}}_{d}\left(\gamma \left(d_{ij}\right)\right)+\max_{k\in K_{i}}\left({\mathrm{MLP}}_{a}\left(\gamma \left(\alpha_{ij}^{k}\right)\right)\right),\;\forall j\in {\hat{N}}_{i},$$

where γ(·) represents the sinusoidal positional encoding, $d_{ij}$ denotes the relative Euclidean distance, and $\alpha_{ij}^{k}$ represents the angular features derived from local triplets. For this angular feature tensor, the subscript *i* represents the center query point serving as the vertex of the spatial angle, while *j* represents the neighbor point and the superscript *k* identifies the specific reference point selected from $K_{i}$ to complete the local triplet. Additionally, the notations ${\mathrm{MLP}}_{d}$ and ${\mathrm{MLP}}_{a}$ denote independent Multi-Layer Perceptron networks specifically designed to process the distance and angular features, respectively. The set $K_{i}\subseteq {\hat{N}}_{i}$ comprises the top-3 nearest neighbors of ${\hat{p}}_{i}$, serving as angular references to construct the local triplets. The max-pooling operator ensures permutation invariance within the angular neighborhood.

We inject the geometric embeddings into the attention mechanism to guide feature aggregation. For each superpoint *i*, attention scores are computed only against its neighbors $j\in {\hat{N}}_{i}$. The key point is that the scope of softmax normalization is constrained to the local neighborhood instead of the entire graph:

(6) $$Q_{i}={\hat{f}}_{i}^{\left(l-1\right)}W^{Q},\;K_{j}={\hat{f}}_{j}^{\left(l-1\right)}W^{K},\;V_{j}={\hat{f}}_{j}^{\left(l-1\right)}W^{V},$$

(7) $$e_{ij}=\frac{Q_{i}{\left(K_{j}+G_{ij}W^{G}\right)}^{⊤}}{\sqrt{d_{k}}},$$

(8) $$a_{ij}=\frac{\exp(e_{ij})}{\sum_{m\in {\hat{N}}_{i}}\exp\left(e_{im}\right)}.$$

Here, $Q_{i}$ represents the query vector derived from the features of the center point *i*, while $K_{j}$ and $V_{j}$ denote the Key and Value vectors generated from the neighbor point *j*.

Finally, the locally enhanced geometric features ${\hat{f}}_{i,\mathrm{local}}^{\left(l\right)}$ are obtained by aggregating the value vectors weighted by the geometry-aware attention scores, followed by a residual connection:

(9) $${\hat{f}}_{i,\mathrm{local}}^{\left(l\right)}={\hat{f}}_{i}^{\left(l-1\right)}+\sum_{j\in {\hat{N}}_{i}}a_{ij}V_{j}.$$

This sparse design not only reduces the computational complexity from $O({\hat{N}}^{2})$ to $O(\hat{N}\times \hat{K})$, where $\hat{K}\ll \hat{N}$, but also forces the network to concentrate on preserving high-fidelity local geometric structures.

#### 3.2.2. Mamba Encoder

Following local enhancement, the features are fed into a Mamba module to capture global context. By leveraging the linear complexity of SSMs, this layer effectively compensates for the limited receptive field of the Local-GSA without incurring the quadratic cost of global Transformers.

Unlike existing Mamba approaches that rely on complex reordering strategies to implicitly preserve structure, HGSAM decouples local geometric anchoring from global sequence modeling. Since the superpoint features have already incorporated local structural information through MG-Conv and Local-GSA, the essential local topology is effectively preserved within the feature channel dimension. Each feature vector functions as a complete geometric descriptor, making the global context propagation process robust to the serialization order. Therefore, we directly serialize the input features $F_{\mathrm{local}}^{\left(l\right)}={\left\{{\hat{f}}_{i,\mathrm{local}}^{\left(l\right)}\right\}}_{i=1}^{\hat{N}}$ into a 1D sequence $X={\left\{x_{t}\right\}}_{t=1}^{\hat{N}}$ following the natural storage order determined by the voxel-based downsampling. This sequence is then processed by Layer Normalization before entering the core Mamba block, which forks into two parallel branches.

In the primary branch, the sequence undergoes linear projection, 1D Depth-Wise Convolution (DWConv), and SiLU activation to yield the intermediate state $X_{\mathrm{ssm}}$. The 1D convolution here acts as a local sequence mixer before the global state space modeling. Subsequently, $X_{\mathrm{ssm}}$ is processed by the discretized selective SSM to propagate global geometry [20]. The state evolution is governed by the following:

(10) $$h_{t}={\bar{A}}_{t}h_{t-1}+{\bar{B}}_{t}x_{\mathrm{ssm},t},\;y_{t}=C_{t}h_{t},$$

where discrete system matrices are derived via the zero-order hold rule with a dynamic timescale $\Delta_{t}$:

(11) $${\bar{A}}_{t}=\exp\left(\Delta_{t}A\right),\;{\bar{B}}_{t}=\left(\exp\left(\Delta_{t}A\right)-I\right)A^{-1}B_{t}.$$

Here, A represents the continuous-state transition matrix that governs the evolution of the hidden state, while $B_{t}$ and $C_{t}$ denote the dynamic continuous input and output projection matrices, respectively. These parameters are strictly optimized during training and are essential for mapping the discrete point cloud sequence into a high-dimensional continuous state space to capture long-range geometric dependencies.

Finally, the output sequence Y from the state space model is fused with the parallel gating branch via element-wise multiplication. The fused feature is then projected through an output linear layer. Since the serialized features maintain strict dimensional alignment with the local features of the 3D point cloud, we directly perform a residual addition equipped with a DropPath mechanism between the projected results and the original local features to obtain the final output:

(12) $$F_{\mathrm{out}}^{\left(l\right)}=\mathrm{Linear}\left(Y⊙\sigma (\mathrm{Linear}(X))\right)+F_{\mathrm{local}}^{\left(l\right)},$$

where ⊙ denotes element-wise multiplication and σ represents the SiLU activation function. This direct residual aggregation mechanism avoids additional computational overhead while ensuring the seamless integration of global sequence modeling and local spatial geometry.

#### 3.2.3. Cross-Attention

While the Mamba Encoder effectively captures intra-cloud global context, establishing robust correspondences requires explicit interaction between the source and target point clouds. To address this, we employ a cross-attention mechanism at the end of the cascading structure. It takes the output features from the preceding Mamba stage as input, which we denote as $F_{\mathrm{out}}^{\hat{P},\left(l\right)}={\left\{{\hat{f}}_{i,\mathrm{out}}^{\left(l\right)}\right\}}_{i=1}^{\hat{N}}$ for the source point cloud and $F_{\mathrm{out}}^{\hat{Q},\left(l\right)}={\left\{{\hat{f}}_{j,\mathrm{out}}^{\left(l\right)}\right\}}_{j=1}^{\hat{M}}$ for the target point cloud. For each source feature ${\hat{f}}_{i,\mathrm{out}}^{\left(l\right)}$, we compute its query vector $q_{i}$, while the key and value vectors $k_{j}$ and $v_{j}$ are derived from every target feature ${\hat{f}}_{j,\mathrm{out}}^{\left(l\right)}$. The global interaction is formulated as

(13) $$e_{ij}=\frac{q_{i}k_{j}^{⊤}}{\sqrt{d_{k}}},\;a_{ij}=\frac{\exp(e_{ij})}{\sum_{m\in \hat{Q}}\exp\left(e_{im}\right)},$$

(14) $${\hat{f}}_{i}^{\left(l\right)}=\sum_{j\in \hat{Q}}a_{ij}v_{j}+{\hat{f}}_{i,\mathrm{out}}^{\left(l\right)}.$$

Similarly, the updated features for the target point cloud ${\hat{f}}_{j}^{\left(l\right)}$ are obtained by treating $\hat{Q}$ as the query and $\hat{P}$ as the key and value sets.

In this formulation, the softmax operation serves as a crucial normalization step that converts raw similarity scores into a valid probability distribution. The purpose of this transformation is to generate relative attention weights that highlight the most highly correlated point pairs between the two clouds. Crucially, the exponential nature of the softmax function inherently amplifies the weights of reliable geometric matches, forcing the network to focus on high-confidence correspondences, which ultimately enhances the overall registration accuracy.

By iteratively integrating Local-GSA, Mamba Encoder, and cross-attention for *L* layers, the output $F^{\hat{P},\left(l\right)}={\left\{{\hat{f}}_{i}^{\left(l\right)}\right\}}_{i=1}^{\hat{N}}$ serves directly as the input for the subsequent layer’s Local-GSA. This hierarchical design yields the final hybrid representations $F^{\hat{P}}=F^{\hat{P},\left(L\right)}$ and $F^{\hat{Q}}=F^{\hat{Q},\left(L\right)}$, enabling our architecture to achieve a comprehensive perception of both local geometry and global context.

### 3.3. Physically Consistent Robust Hypothesis Proposer

Following the context aggregation stage, we obtain the hybrid source features $F_{P}\in R^{\hat{N}\times C}$ and target features $F_{Q}\in R^{\hat{M}\times C}$. To determine reliable superpoint correspondences, we first compute a similarity matrix $S\in R^{\hat{N}\times \hat{M}}$ between $F_{P}$ and $F_{Q}$ utilizing a Gaussian correlation function. Subsequently, to suppress ambiguous associations and enhance distinctiveness, a dual-normalization operation is performed on the correlation matrix S. As reported in [48], this normalization step effectively filters out outliers by suppressing weights in rows and columns with multiple high responses.

**Figure 5:**
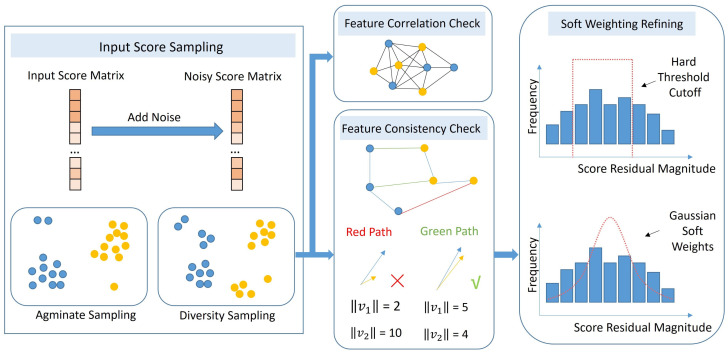
Overview of the proposed PCRH-P. The blue and yellow circles represent points from the source and target point clouds respectively. The red path and red cross indicate inconsistent feature matches while the green path and green check mark denote consistent ones.

Given this refined similarity matrix S, the subsequent step is to extract a set of discrete putative correspondences for the final rigid pose estimation. While conventional methods typically rely on RANSAC or standard top-*k* sampling to form the correspondence set, these approaches often suffer from spatial clustering, where hypotheses are disproportionately drawn from high-confidence local regions. This tendency leads to a neglect of global physical consistency and entrapment in local optima. To overcome these limitations and secure a highly reliable transformation, we introduce the PCRH-P, as illustrated in Figure 5.

#### 3.3.1. Spatial Diversity Sampling

To encourage a broader distribution of samples, we introduce a spatial diversity sampling strategy during the superpoint matching phase. Instead of strictly selecting matches based on the original scores, we inject stochastic noise ζ into the score matrix prior to selection to disrupt the tie-breaking of similar scores and disperse the sampling distribution:

(15) $$S_{ij}^{\prime }=S_{ij}+\zeta_{ij},\;\zeta_{ij}∼U\left(0,\xi \right),$$

where ξ is a small constant (e.g., $10^{-4}$). Finally, the robust superpoint correspondence set $\hat{G}$ is established by selecting the top-*k* entries based on the perturbed scores:

(16) $$\hat{G}=\left\{\left({\hat{p}}_{i},{\hat{q}}_{j}\right)|\left(i,j\right)\in {\mathrm{Top}}_{k}\left(S^{\prime }\right)\right\},$$

where ${\mathrm{Top}}_{k}\left(\cdot \right)$ denotes the operation of extracting the set of index pairs (i,j) corresponding to the *k* largest entries in the score matrix $S^{\prime }$. This strategy ensures that the generated hypotheses maintain both spatial distinctiveness and structural integrity.

#### 3.3.2. Point Matching

Upon establishing the reliable superpoint correspondence set $\hat{G}$ via spatial diversity sampling, we extend these coarse matches to the fine-grained level. Following the point-to-node grouping strategy [37], we assign dense points $\tilde{P}$ to their corresponding superpoints $\hat{P}$ by leveraging the nearest-neighbor upsampling indices. The sparse superpoints are mapped back to their high-resolution dense counterparts to reconstruct local dense patches, denoted as ${\tilde{P}}_{x}$ and ${\tilde{Q}}_{y}$. This process yields the dense point coordinates along with their associated rotation-invariant features $F_{x}^{\tilde{P}}$, $F_{y}^{\tilde{Q}}$ and rotation-equivariant features $E_{x}^{\tilde{P}}$, $E_{y}^{\tilde{Q}}$. For the points within each local patch, we compute the feature correlation scores to establish fine-grained correspondences. We first compute the initial local similarity matrix $Z_{x,y}$ using the inner product of the projected features:

(17) $$Z_{x,y}=\frac{{\left(W_{m}F_{x}^{\tilde{P}}\right)}^{⊤}\left(W_{m}F_{y}^{\tilde{Q}}\right)}{\sqrt{3\tilde{d}}},$$

where $W_{m}$ is a learnable projection matrix, and $\tilde{d}$ denotes the feature dimension. Then, we calculate the saliency scores ψ for each point using a Sigmoid-activated projection:

(18) $$\psi_{x}^{\tilde{P}}=\mathrm{Sigmoid}\left(W_{\psi }F_{x}^{\tilde{P}}\right).$$

The final dense matching score matrix $\tilde{S}$ is then derived by combining these saliency scores with a dual-normalization operation. This step effectively filters out outliers by performing softmax normalization across both rows and columns of the local similarity matrix:

(19) $${\tilde{S}}_{x,y}=\psi_{x}^{\tilde{P}}⊙\psi_{y}^{\tilde{Q}}⊙{\mathrm{Softmax}}_{\mathrm{row}}\left(Z_{x,y}\right)⊙{\mathrm{Softmax}}_{\mathrm{col}}\left(Z_{x,y}\right).$$

To balance hypothesis generation efficiency with geometric verification robustness, we construct two functionally distinct correspondence subsets based on these scores: a high-confidence set ${\tilde{C}}_{\mathrm{init}}=\left\{\left({\tilde{x}}_{i},{\tilde{y}}_{j}\right)\right\}$ selected via global top-*k* for hypothesis generation, and a broader voting set ${\tilde{C}}_{\mathrm{valid}}=\left\{\left({\tilde{p}}_{u},{\tilde{q}}_{v}\right)\right\}$ determined by a thresholded bidirectional top-*k* strategy to ensure high inlier recall for robust geometric verification.

#### 3.3.3. Loss Function

Following the supervision paradigm established in [30], our loss function is composed of three complementary components. The network is trained by minimizing the total loss L, which is a summation of the coarse-level superpoint matching term $L_{c}$, the fine-level point matching term $L_{f}$, and the rotational equivariance regularization term $L_{r}$. Here, $L_{c}$ and $L_{f}$ guarantee the quality of coarse-to-fine correspondences, while $L_{r}$ enhances feature robustness against arbitrary rotations:

(20) $$L=L_{c}+L_{f}+L_{r}.$$

Specifically, the coarse-level loss $L_{c}$ supervises the matching quality between the source superpoints $\hat{P}$ and the target superpoints $\hat{Q}$. We adopt a circle loss with a reweighting mechanism to enhance the distinctiveness of superpoint features. For each source superpoint *i*, we construct a positive sample set $E_{p}$ and a negative sample set $E_{n}$ according to their spatial overlap ratios. The formulation is defined as follows:

(21) $$L_{c}=\frac{1}{|\hat{P}|}\sum_{i\in \hat{P}}\log\left(1+\sum_{j\in E_{p}}\exp\left(\gamma \alpha_{ij}^{p}\left(d_{ij}-\Delta_{p}\right)\right)\sum_{k\in E_{n}}\exp\left(\gamma \alpha_{ik}^{n}\left(\Delta_{n}-d_{ik}\right)\right)\right),$$

where $d_{ij}$ and $d_{ik}$ denote the Euclidean distances in the feature space for positive and negative pairs, respectively. γ represents the scale factor. $\Delta_{p}$ and $\Delta_{n}$ denote the margin thresholds used to demarcate positive and negative samples. $\alpha_{ij}^{p}$ and $\alpha_{ik}^{n}$ are adaptive weighting coefficients adjusted based on feature distances.

The fine-level loss $L_{f}$ targets the dense point clouds $\tilde{P}$ and $\tilde{Q}$. Within the local overlapping regions determined by the coarse correspondences, the network predicts a soft matching probability matrix $\tilde{S}$. We compute this cross-entropy loss by minimizing the negative log-likelihood of the ground-truth matching point pairs $M_{gt}$:

(22) $$L_{f}=-\frac{1}{|M_{gt}|}\sum_{\left(x,y\right)\in M_{gt}}\log{\tilde{S}}_{x,y},$$

where ${\tilde{S}}_{x,y}$ indicates the predicted probability of establishing a correct match between dense points *x* and *y*.

The regularization term enforces the commutativity between spatial rotation and feature extraction:

(23) $$L_{r}=\frac{1}{\left|P\right|}\sum_{i=1}^{\left|P\right|}{∥f\left(RP_{i}\right)-Rf\left(P_{i}\right)∥}_{2}^{2},$$

where f(·) is the feature extraction operation, $P_{i}$ is the input local point cloud patch comprsing the spatial neighborhood, and R is an arbitrary 3D rigid rotation matrix. This formulation minimizes the mathematical deviation under any initial pose, ensuring that the network maintains strict equivariance throughout the geometric encoding process.

#### 3.3.4. Feature Norm Consistency Pruning

A fundamental physical invariant of rigid transformations is length preservation. Since our MG-Conv backbone generates rotation-equivariant features, their Frobenius norms are mathematically invariant to rotation (i.e., ${∥Rf∥}_{F}={∥f∥}_{F}$). We leverage this strong physical prior to design an early feature norm consistency pruning strategy. For every initial correspondence $\left(x_{i},y_{j}\right)\in {\tilde{C}}_{\mathrm{init}}$, we quantify the violation of this geometric constraint via the relative feature norm discrepancy $\delta_{ij}$:

(24) $$\delta_{ij}=\frac{\left|∥E_{x_{i}}^{\tilde{P}}{∥}_{F}-{∥E_{y_{j}}^{\tilde{Q}}∥}_{F}\right|}{\max(∥E_{x_{i}}^{\tilde{P}}{∥}_{F},∥E_{y_{j}}^{\tilde{Q}}{∥}_{F})},$$

where $E_{x_{i}}^{\tilde{P}}\in R^{C\times 3}$ and $E_{y_{j}}^{\tilde{Q}}\in R^{C\times 3}$ denote the dense rotation-equivariant feature tensors for the source and target points, respectively. The operator ${∥\cdot ∥}_{F}$ represents the Frobenius norm calculated across the feature dimensions. In this formulation, $\delta_{ij}$ serves as a normalized residual that measures the structural deviation between paired features. Correspondences satisfying $\delta_{ij}>\tau_{\mathrm{norm}}$ are immediately pruned, where $\tau_{\mathrm{norm}}$ is a predefined consistency threshold. This mechanism filters out outliers that may possess high matching scores but violate the intrinsic rigidity of the point cloud.

#### 3.3.5. Gaussian Soft-Weighted Refinement

Utilizing the pruned set ${\tilde{C}}_{\mathrm{init}}$, we generate a diverse pool of transformation hypotheses $H=\{T_{k}\}$. Since each rotation-equivariant feature matrix $E\in R^{C\times 3}$ encapsulates the local geometric orientation, the rotation $R_{k}$ can be estimated by aligning the feature columns of a matched pair $\left(x_{i},y_{j}\right)\in {\tilde{C}}_{\mathrm{init}}$ via SVD:

(25) $$R_{k}=\underset{R\in SO(3)}{arg min}{∥R{\left(E_{x_{i}}^{\tilde{P}}\right)}^{⊤}-{\left(E_{y_{j}}^{\tilde{Q}}\right)}^{⊤}∥}_{F}^{2}.$$

Subsequently, the translation $t_{k}$ is recovered using the point coordinates:

(26) $$t_{k}=y_{j}-R_{k}x_{i}.$$

A significant advantage of this formulation is that it enables single correspondence pose estimation. Because the rotation equivariant feature matrices inherently capture the complete local reference frame, the full six degrees of freedom transformation can be uniquely determined from just one matched pair. This fundamentally differs from traditional estimators that require at least three pairs, drastically reducing the combinatorial search space for generating hypotheses.

Finally, we perform comprehensive validation and refinement of the obtained hypothesis pool H using the broader voting set ${\tilde{C}}_{\mathrm{valid}}$. The correspondence pairs $\left({\tilde{p}}_{u},{\tilde{q}}_{v}\right)\in {\tilde{C}}_{\mathrm{valid}}$ utilized in this stage represent the high-resolution spatial coordinates extracted from the foundational layers of the hierarchical pyramid backbone. We apply each hypothesis $T_{k}\in H$ to the source points ${\tilde{p}}_{u}$ and compute the spatial residuals against the target points ${\tilde{q}}_{v}$.

In order to provide a more flexible and differentiable alternative to sensitive hard-thresholding mechanisms, we employ a Gaussian soft-weighting strategy. For each correspondence, the weight $w_{uv}$ for a pair $\left({\tilde{p}}_{u},{\tilde{q}}_{v}\right)$ is formulated as a Gaussian kernel:

(27) $$w_{uv}=\exp\left(-\frac{∥T_{k}{\tilde{p}}_{u}-{\tilde{q}}_{v}{∥}_{2}^{2}}{2\omega^{2}}\right),\;\omega =\tau_{accept}/2,$$

where ω controls the sensitivity to outliers. The refinement is then posed as a weighted Procrustes problem, solved iteratively to minimize the soft-weighted objective:

(28) $$T_{\mathrm{final}}=\underset{T}{\arg\min}\sum_{\left({\tilde{p}}_{u},{\tilde{q}}_{v}\right)\in {\tilde{C}}_{\mathrm{valid}}}w_{uv}{∥T{\tilde{p}}_{u}-{\tilde{q}}_{v}∥}_{2}^{2}.$$

We iterate this re-weighting and solving process for $L_{rs}$ steps. This continuous weighting scheme ensures a smooth gradient flow during training and progressively suppresses outliers, yielding a robust and precise alignment $T_{\mathrm{final}}$.

## 4. Results

In this section, we conduct extensive experiments on both indoor and outdoor benchmarks to comprehensively evaluate the performance of the proposed method. To ensure a fair comparison and reproducibility, we implement our model using PyTorch 2.0.1 and train it entirely from scratch, following standard protocols and without leveraging any pre-trained weights. We compare our method with a wide range of recent competitive baselines to demonstrate its superior performance. Furthermore, we provide ablation studies to validate the effectiveness of each proposed component, along with qualitative visualizations to offer intuitive insights into the registration results.

### 4.1. Implementation Details

All experiments are conducted on a workstation equipped with an Intel Xeon Gold 6226 CPU and four NVIDIA V100 GPUs. The framework is implemented using PyTorch 2.0.1, Python 3.8, and CUDA 11.8. The network is optimized using the Adam optimizer with a batch size of 1 and an initial learning rate of $10^{-5}$. As summarized in Table 1, we follow the standard momentum configurations with $\beta_{1}=0.9$ and $\beta_{2}=0.999$. On the 3DMatch dataset, we train for 40 epochs with the learning rate exponentially decayed by a factor of 0.95 after each epoch. For the KITTI dataset, the training duration is extended to 110 epochs, with an initial learning rate of $10^{-4}$ and the learning rate scaled by a factor of 0.95 every 4 epochs. The gradient clipping is not applied during the training phase to maintain the original gradient flow of the Mamba layers.

### 4.2. Indoor Benchmark: 3DMatch and 3DLoMatch

We utilize the standard 3DMatch and 3DLoMatch benchmarks [49], which comprise RGB-D reconstruction data collected from 62 distinct indoor scenes. These scenes encompass diverse indoor environments, such as offices, bedrooms, and living rooms. The ground-truth rigid transformations for this dataset are established by aligning reconstruction fragments into a unified coordinate system. Due to varying scanning perspectives, the collected data exhibit significant distribution shifts and complex occlusion patterns. Furthermore, the prevalent repetitive structures and weak-textured regions in these scenes pose severe challenges for robust point cloud registration. Following the protocols established in [30,33,37], we split the dataset into 46 scenes for training, 8 for validation, and 8 for testing. To rigorously assess the model under varying conditions, the test set is categorized into two subsets based on the overlap ratio of point cloud pairs. 3DMatch contains pairs with an overlap ratio greater than 30%, primarily used to evaluate registration performance under standard conditions. 3DLoMatch contains pairs with an overlap ratio between 10% and 30%.

#### 4.2.1. Evaluation Metrics for Indoor Benchmarks

Following the standard evaluation protocols established in GeoTransformer [37], a comprehensive quantitative analysis is conducted using three standard metrics: Inlier Ratio (IR), Feature Matching Recall (FMR), and Registration Recall (RR). IR is defined as the percentage of putative correspondences whose Euclidean spatial residuals are below a strict threshold with respect to the ground truth. Specifically, a predicted correspondence is considered an inlier if the distance between the aligned points is less than the acceptance radius of 0.1 m. FMR denotes the percentage of point cloud pairs that achieve an inlier ratio exceeding 5%. RR serves as the comprehensive indicator of the final alignment success rate. We classify a registration attempt as successful if the Root Mean Squared Error (RMSE) of the ground-truth correspondences is less than 0.2 m after applying the estimated transformation. This metric reflects the end-to-end performance of the registration pipeline.

#### 4.2.2. Registration Results for Indoor Benchmarks

We benchmark the correspondence quality and registration accuracy of our proposed method against seven recent competitive methods, including PARENet [30], FCGF [8], D3Feat [50], GeoTransformer [37], Predator [33], YOHO [16], and CoFiNet [34]. Adhering to the standard evaluation protocols established in GeoTransformer [37] and Predator [33], we assess performance across varying correspondence budgets. Specifically, the number of correspondences is controlled by modulating the hyperparameter *k* to select the top-ranked matches based on confidence. As *k* decreases from 5000 to 250, the task becomes significantly more challenging, placing higher demands on the distinctiveness of the extracted features.

As shown in Table 2, our method delivers competitive results compared to existing baselines using the RANSAC estimator. On the 3DMatch dataset, our approach secures leading performance in RR, achieving 94.2% with 5000 points. Notably, the model maintains a high RR of 92.9% even when the number of correspondences is significantly reduced to 250, demonstrating superior stability in sparse data conditions.

An objective comparison across the data metrics reveals that GeoTransformer maintains a lead in FMR on the 3DLoMatch dataset and exhibits the highest IR under extremely sparse sample configurations, namely at 500 and 250 sampled points on both datasets. This indicates that GeoTransformer is capable of preserving a remarkably high proportion of correct matches when processing extremely sparse inputs.

However, a higher IR does not completely translate into the success rate of the final registration. Under the 250-point setting, although the IR of our method is lower than that of GeoTransformer, our RR reaches 92.9% on 3DMatch and 73.3% on 3DLoMatch, both of which are higher than the baseline methods. This data phenomenon indicates that the feature matches extracted by our method can highly effectively support the RANSAC algorithm in completing the final pose estimation.

On the more challenging 3DLoMatch dataset, our method achieves a peak RR of 76.0%, demonstrating strong robustness. Synthesizing the data in Table 2, while no single method exerts absolute dominance across all metrics, our approach exhibits significant advantages and high reliability in RR, which is the core indicator of registration success.

In the RANSAC-free setting, we evaluate the full registration pipelines of various methods. As reported in Table 3, these comparisons involve different end-to-end pipelines utilizing distinct back-end estimators such as LGR for GeoTrans and FHP for PARENet, whereas our method employs the PCRH-P solver. Consequently the reported performance gains reflect the synergistic effect between the learned feature representations and their respective back-end solvers rather than a direct feature-to-feature comparison. Benefiting from the efficient geometric structure encoding of the network coupled with the strict physical consistency verification enforced by the PCRH-P mechanism, our method achieves a remarkable registration recall of 96.3%.

As summarized in Table 4, we evaluate the computational efficiency of our proposed framework. Here, the theoretical complexity is measured by GFLOPs, which represents the billion floating-point operations required for a single forward pass, reflecting the intrinsic complexity of the algorithm. Model time refers to the pure computational duration of the front-end feature extraction and interaction network, while pose time refers to the duration for the back-end solver to calculate the final transformation matrix. Peak memory represents the maximum instantaneous GPU memory occupied during inference, and throughput indicates the number of point cloud pairs the system can process per second.

Experimental results show that the theoretical complexity advantage of this study is significant. Our model consumes only 14.482 GFLOPs, which is approximately 9.3% of GeoTransformer and 21.4% of PARENet. This result directly validates the effectiveness of the HGSAM module in reducing point cloud processing complexity from quadratic to linear through local geometric self-attention and the Mamba architecture. Regarding inference latency, although the current model time of 658.20 ms is higher than dense attention methods, it is primarily attributed to the lack of low-level kernel fusion optimization for sparse grouping operators in the PyTorch framework. However, the remarkably low theoretical complexity and peak memory of 3.63 GB signify superior scalability for large-scale point clouds, effectively avoiding out-of-memory errors. Furthermore, by decoupling model inference time and pose estimation time, we find that the PCRH-P backend consumes 15.35 ms, which is in the same order of magnitude as the competing methods. This evidence strongly demonstrates that the 96.3% registration recall achieved on 3DMatch is primarily due to the high-quality features extracted by the front-end, rather than solely relying on the computational contribution of the back-end.

As reported in Table 5, we quantitatively compare our proposed method with three baseline models. The results indicate that our method consistently outperforms the competitors in most scenes. Notably, our method achieves perfect registration in the Hotel_2 and Hotel_3 scenes. Furthermore, in the challenging MIT_Lab scene, our method attains a recall of 97.8%, which highlights the model’s capability in handling complex structures.

#### 4.2.3. Qualitative Visualization

**Figure 6:**
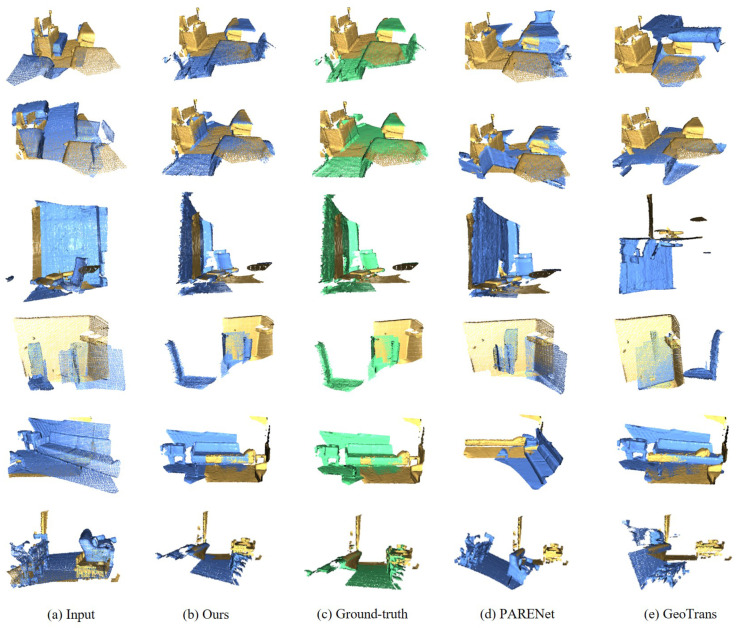
Qualitative results, where the source point cloud is denoted in yellow, the target point cloud in blue, and the ground-truth in green. It displays the visualization of the predicted registration results.

**Figure 7:**
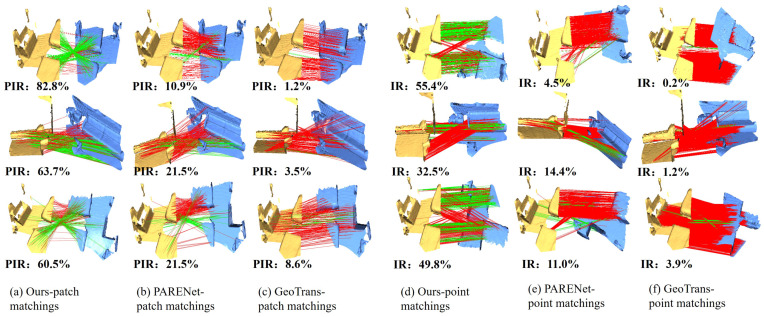
Our approach successfully aligns low-overlap pairs, outperforming competitors in both IR and PIR. It illustrates the correctness of the correspondences identified during both the coarse superpoint matching stage on the left and the fine-level registration stage on the right. The yellow and blue structures represent the source and target point clouds respectively. The green lines indicate correct correspondences while the red lines denote incorrect matches.

To provide a more intuitive demonstration of the registration performance achieved by our proposed algorithm, we present qualitative visualizations on the 3DMatch and 3DLoMatch test sets in Figure 6 and Figure 7.

Figure 6 presents a qualitative comparison of our method against GeoTrans [37] and PARENet [30] in challenging scenarios. As observed, PARENet suffers from ambiguous feature matching on prominent features due to insufficient geometric cues, leading to local optima and significant rotational errors that fail to correctly align the source and target point clouds. Similarly, GeoTrans still exhibits limitations in registering planar regions, resulting in noticeable misalignments. In contrast, our method achieves superior alignment accuracy, visually indistinguishable from the ground truth. This is attributable to the injection of explicit high-order local geometric cues into rotation-equivariant features, which effectively mitigates geometric ambiguities and guarantees robust and precise registration.

To investigate the underlying mechanism of the superior performance, we visualize the coarse-level and fine-level correspondences in Figure 7. At the coarse level, existing methods generate numerous erroneous connections, which are disproportionately clustered in local regions, leading to spatial degeneracy. Conversely, benefitting from the spatial diversity sampling strategy, our method yields a spatially uniform distribution of correspondences with significantly fewer outliers. At the fine level, our approach maintains a higher density of correct matches on the overlapping surfaces. We quantify this local matching quality using the Patch Inlier Ratio (PIR), defined as the fraction of inlier correspondences within a local patch relative to the total number of retrieved correspondences. The significantly improved PIR validates that the feature norm consistency pruning module effectively filters out distinct but geometrically inconsistent pairs, providing a high-quality correspondence set for the subsequent pose estimation.

### 4.3. Outdoor Benchmark: KITTI

We employ the large-scale KITTI odometry dataset [51] to test our proposed method’s effectiveness for outdoor scenes, which consists of 11 autonomous driving sequences characterized by sparse LiDAR point cloud distributions. The reference ground truth for these sequences is established using a high-precision GPS/IMU navigation system coupled with specialized calibration. Unlike dense indoor data, KITTI point clouds are distributed over large-scale spatial areas, testing the model’s capacity to aggregate long-range geometric dependencies. Following the data-splitting configuration used in GeoTransformer [37], we use sequences 00–05 for training, 06–07 for validation, and 08–10 for testing.

#### 4.3.1. Evaluation Metrics for Outdoor Benchmarks

We adopt the standard evaluation protocols from [37] to validate our model, utilizing three specific metrics. We calculate the Relative Rotation Error (RRE) as the geodesic distance between the estimated rotation and the ground truth rotation. The Relative Translation Error (RTE) is defined as the Euclidean distance between the translation vectors. Finally, we report the RR, which indicates the fraction of successful registrations where both errors remain within specific limits (RRE <5° and RTE <2 m).

#### 4.3.2. Registration Results for Outdoor Benchmarks

**Figure 8:**
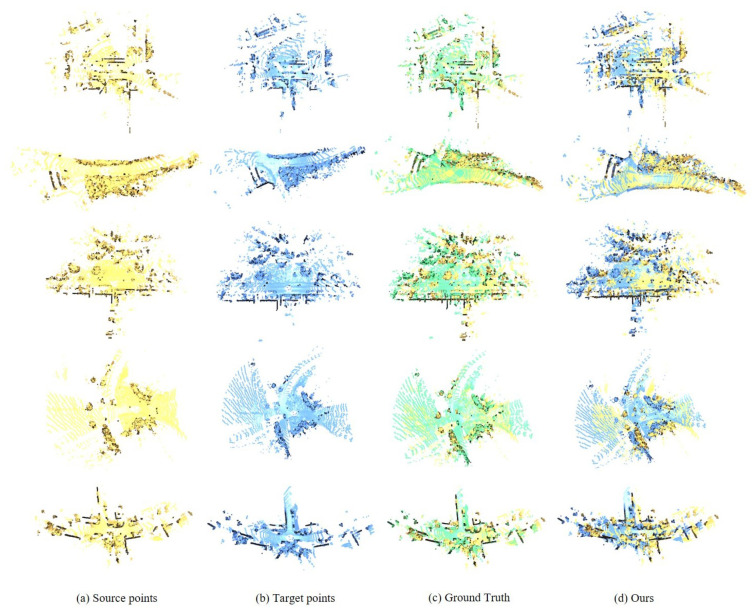
Qualitative results on the KITTI Odometry dataset, where the source point cloud is denoted in yellow, the target point cloud in blue, and the ground-truth in green.

We report the quantitative results on the KITTI dataset in Table 6. While high registration recall has become common on the KITTI benchmark, achieving low-drift alignment remains challenging. Our method distinguishes itself by achieving the lowest registration errors, as well as the lowest rotation and translation errors among all compared methods. In addition, some qualitative results are shown in Figure 8.

### 4.4. Ablation Study

To validate the effectiveness of each proposed component, we conduct a comprehensive ablation study on the 3DMatch dataset. As summarized in Table 7, we establish a baseline model and incrementally integrate the MG-Conv, HGSAM, and PCRH-P.

The baseline model employs standard rotation-equivariant convolution operators PARE-Conv that rely primarily on spatial coordinates. By replacing the backbone with our MG-Conv (Model B), we observe a steady improvement in PIR, rising from 84.1% to 85.3%, validating that the explicit encoding of multivariate geometric features enables MG-Conv to extract more discriminative local descriptors. This enhances the robustness of patch-level feature matching, providing a stable foundation for subsequent global aggregation.

To explore the relationship between computational efficiency and architectural design, we compare Model C and Model E. Model C attempts to enhance global perception by stacking two standard self-attention modules, which increases the inference time to 0.649 s due to its quadratic complexity constraints. In contrast, after introducing the HGSAM module with linear complexity (Model E), the model significantly compresses the time consumption to 0.190 s while obtaining better performance. This result strongly demonstrates that HGSAM can effectively mitigate the computational burden of traditional Transformer architectures during global modeling.

Furthermore, we directly incorporate the HGSAM into the baseline (Model D), observing a significant improvement in the IR. This result confirms its strong capability to effectively focus on overlapping regions during feature interaction. Furthermore, combining HGSAM with MG-Conv to form Model E not only increases PIR and IR but also boosts registration recall to 95.8%, outperforming both the baseline and Model B. The MG-Conv provides discriminative local geometric features, while the HGSAM aggregates these features over long ranges. This result validates that the cooperation between local geometry and global perception effectively resolves ambiguities in repetitive structures that local convolutions alone cannot address.

Finally, we incorporate the PCRH-P (Model F) to replace the standard feature hypothesis estimator. The PCRH-P ensures that the generated hypotheses are not only high-confidence but also geometrically plausible, resulting in a more precise final alignment. This yields the highest overall registration success, pushing the registration recall to a peak of 96.3% while maintaining an inference time of 0.192 s, proving the contribution of an efficient back-end to robustness without sacrificing speed.

## 5. Conclusions

In this paper, we present a robust point cloud registration framework driven by multivariate geometric perception. To address the inherent ambiguity in scenes with repetitive structures and weak textures in point cloud registration tasks, our method effectively enhances the perception of fine topological details by integrating explicit physical invariants into rotation-equivariant feature extraction via the MGCE mechanism. Furthermore, the proposed HGSAM successfully establishes a connection between explicit physical awareness and efficient global context modeling, utilizing a cascade of local geometric attention and Mamba’s selective scan mechanism. The spatial diversity sampling, consistency pruning and soft-weighted refinement within the PCRH-P yield a substantial improvement in alignment reliability, while decoupled efficiency analysis shows that this back-end ensures robustness without adding significant computational burden. Extensive experiments show that our framework is highly effective for registration in challenging large-scale scenarios. Regarding computational efficiency, although our method validates the theoretical advantage of linear complexity with only 14.482 GFLOPs, its actual inference latency still faces limitations on resource-constrained edge devices. This is primarily due to the fact that the current PyTorch implementation has yet to fully leverage the potential of the Mamba architecture for low-level hardware acceleration. Future research will focus on model lightweighting and specialized kernel optimization, while further exploring multi-view registration strategies to achieve improved global mapping.

## Figures and Tables

**Table 1:** Detailed hyperparameter settings for the proposed method across different modules.

Category	Parameter	Symbol	Value
Architecture	Neighborhood Size	*K*	35
	Initial Voxel Size	$v_{size}$	0.025 m
	Feature Dimensions	$d_{init}/d_{out}$	32/256
HGSAM	Neighborhood Size	$\hat{K}$	$\min(35,|\hat{P}|)$
	Number of Iterations	*L*	3
	Number of Attention Heads	*h*	4
	Input Dimension	$d_{in}$	768
	Output Dimension	$d_{k}$	192
PCRH-P	Perturbation Magnitude	ξ	$1\times 10^{-4}$
	Pruning Threshold	$\tau_{norm}$	0.08
	Acceptance Radius	$\tau_{accept}$	0.1 m
	Number of Hypotheses	|H|	1000
	Refinement Steps	$L_{rs}$	5
Optimizer	Adam Hyperparameters	$\beta_{1},\beta_{2},\epsilon$	0.9, 0.999, $10^{-8}$
	Weight Decay	-	$1\times 10^{-6}$
	Learning Rate Decay	-	0.95
	Fixed Random Seed	-	7351

**Table 2:** Performance comparison on 3DMatch and 3DLoMatch datasets. RANSAC [21] is used for registration with 50 K iterations. **Bold** and underlined values indicate the best and second-best results, respectively. ↑ indicates that higher values are better.

Method	3DMatch					3DLoMatch				
	5000	2500	1000	500	250	5000	2500	1000	500	250
*Feature Matching Recall (%) ↑*										
FCGF [8]	97.4	97.3	97.0	96.7	96.6	76.6	75.4	74.2	71.7	67.3
D3Feat [50]	95.6	95.4	94.5	94.1	93.1	67.3	66.7	67.0	66.7	66.5
Predator [33]	96.6	96.6	96.5	96.3	96.5	78.6	77.4	76.3	75.7	75.3
YOHO [16]	98.2	97.6	97.5	97.7	96.0	79.4	78.1	76.3	73.8	69.1
CoFiNet [34]	98.1	98.3	98.1	98.2	98.3	83.1	83.5	83.3	83.1	82.6
GeoTransformer [37]	97.9	97.9	97.9	97.9	97.6	**88.3**	**88.6**	**88.8**	**88.6**	**88.3**
PARENet [30]	98.5	98.5	98.5	98.5	98.7	87.3	87.3	87.3	87.4	87.1
Ours	**99.0**	**99.0**	**99.0**	**98.8**	**98.8**	88.2	88.2	88.2	88.0	87.8
*Inlier Ratio (%) ↑*										
FCGF [8]	56.8	54.1	48.7	42.5	34.1	21.4	20.0	17.2	14.8	11.6
D3Feat [50]	39.0	38.8	40.4	41.5	51.8	13.2	13.1	14.0	14.6	15.0
Predator [33]	58.0	58.4	57.1	54.1	49.3	26.7	28.1	28.3	27.5	25.8
YOHO [16]	64.4	60.7	55.7	46.4	41.2	25.9	23.3	22.6	18.2	15.0
CoFiNet [34]	49.8	42.2	51.9	52.2	52.2	24.4	25.9	26.7	26.8	26.9
GeoTransformer [37]	71.9	75.2	76.0	**82.2**	**85.1**	43.5	45.3	46.2	**52.9**	**57.7**
PARENet [30]	75.3	75.3	75.3	77.7	79.4	45.2	45.3	45.3	47.6	49.3
Ours	**76.9**	**76.9**	**76.9**	79.2	80.7	**47.2**	**47.2**	**47.2**	49.3	51.3
*Registration Recall (%) ↑*										
FCGF [8]	85.1	84.7	83.3	81.6	71.4	40.1	41.7	38.2	35.4	26.8
D3Feat [50]	81.6	84.5	83.4	82.4	77.9	37.2	42.7	46.9	43.8	39.1
Predator [33]	89.0	89.9	90.6	88.5	86.6	59.8	61.2	62.4	60.8	58.1
YOHO [16]	90.2	90.3	89.1	88.6	84.5	65.2	65.5	63.2	56.5	48.0
CoFiNet [34]	89.3	88.9	88.4	87.4	87.0	67.5	66.2	64.2	63.1	61.0
GeoTransformer [37]	92.0	91.8	91.8	91.4	91.2	75.0	74.8	72.2	74.1	73.5
PARENet [30]	92.5	92.9	92.7	91.8	91.4	75.1	74.9	73.2	74.3	71.8
Ours	**94.2**	**94.1**	**93.4**	**93.0**	**92.9**	**75.9**	**76.0**	**75.7**	**74.8**	**73.3**

**Table 3:** Registration results without RANSAC on 3DMatch. **Bold** indicates the best results. ↑ indicates that higher values are better. ↓ indicates that lower values are better.

Model	Estimator	Samples	Size (MB)	RR (%) ↑	RRE (°) ↓	RTE (m) ↓
FCGF [8]	RANSAC-50k	5000	8.76	87.6	–	–
RoReg [15]	RANSAC-50k	5000	10.06	93.0	–	–
Predator [33]	LGR	all	7.43	89.0	2.029	0.064
GeoTrans [37]	LGR	all	9.83	92.5	1.772	**0.061**
PARENet [30]	FHP	all	3.84	95.0	1.888	0.062
Ours	PCRH-P	all	5.40	**96.3**	**1.705**	0.062

**Table 4:** Efficiency comparison on the 3DMatch dataset. We report theoretical complexity measured in GFLOPs, inference latency, and hardware resource consumption. **Bold** values indicate the best results. ↑ indicates higher is better; ↓ indicates lower is better.

Model	GFLOPs ↓	Model Time (ms) ↓	Pose Time (ms) ↓	Peak Mem (GB) ↓	Throughput (p/s) ↑
GeoTrans [37]	154.614	**351.87**	**13.75**	3.81	**2.84**
PARENet [30]	67.562	445.55	15.10	**3.59**	2.24
**Ours**	**14.482**	658.20	15.35	3.63	1.52

**Table 5:** Comparison of results across different scenes on 3DMatch. **Bold** values indicate the best results. ↑ indicates higher is better and ↓ indicates lower is better.

Model	Kitchen	Home_1	Home_2	Hotel_1	Hotel_2	Hotel_3	Study	MIT_Lab	Mean
*Registration Recall (%) ↑*									
Predator [33]	97.9	97.2	74.5	98.5	96.2	88.6	86.1	73.4	89.1
GeoTransformer [37]	98.2	**98.1**	83.6	97.8	92.3	88.5	90.2	91.1	92.5
PARENet [30]	**99.6**	**98.1**	**85.5**	**99.5**	97.4	92.3	88.5	93.3	94.3
Ours	99.1	**98.1**	**85.5**	98.9	**100.0**	**100.0**	**90.6**	**97.8**	**96.3**
*Rotation Error (°) ↓*									
Predator [33]	1.861	1.806	2.473	2.045	1.600	2.458	2.067	1.926	2.029
GeoTransformer [37]	**1.829**	1.534	2.076	1.569	1.553	1.715	1.914	1.986	1.772
PARENet [30]	2.482	1.598	2.253	1.641	1.632	**1.628**	2.105	1.765	1.888
Ours	2.304	**1.371**	**1.995**	**1.439**	**1.328**	1.685	**1.876**	**1.647**	**1.705**
*Translation Error (m) ↓*									
Predator [33]	0.048	0.055	0.070	0.073	**0.060**	0.065	0.080	**0.063**	0.064
GeoTransformer [37]	0.047	0.052	**0.062**	**0.057**	0.061	0.051	0.080	0.078	**0.061**
PARENet [30]	0.043	**0.049**	0.082	0.058	**0.060**	**0.048**	**0.079**	0.073	0.062
Ours	**0.040**	0.051	0.080	0.059	**0.060**	0.053	0.081	0.075	0.062

**Table 6:** Registration results without RANSAC on KITTI. **Bold** values indicate the best results. ↑ indicates that higher values are better. ↓ indicates that lower values are better.

Model	Estimator	Size (MB)	RR (%) ↑	RRE (°) ↓	RTE (cm) ↓
FCGF [8]	RANSAC-50k	8.76	96.6	0.30	9.5
D3Feat [50]	RANSAC-50k	14.08	**98.8**	0.30	7.2
Predator [33]	LGR	22.77	**99.8**	0.27	6.8
CoFiNet [34]	LGR	5.48	**99.8**	0.41	8.2
GeoTransformer [37]	LGR	25.50	99.8	0.27	6.8
Ours	PCRH-P	**2.53**	**99.8**	**0.22**	**5.2**

**Table 7:** Ablation experiments on 3DMatch. We compare the backbone using different convolution operators, interaction blocks, and pose estimation strategies. **Bold** values indicate the best results. ↑ indicates that higher values are better. ↓ indicates that lower values are better.

Model	Backbone	Feature Interaction	Estimator	PIR (%) ↑	FMR (%) ↑	IR (%) ↑	RR (%) ↑	Time (s) ↓
Model A	PARE-Conv	Self + Cross	FHP	84.1	98.4	72.0	94.3	**0.165**
Model B	MG-Conv	Self + Cross	FHP	85.3^+1.2^	98.6^+0.2^	73.6^+1.6^	94.9^+0.6^	0.176
Model C	MG-Conv	Self + Self + Cross	FHP	**85.4^+1.3^**	98.6^+0.2^	75.4^+3.4^	95.0^+0.7^	0.649
Model D	PARE-Conv	HGSAM	FHP	84.8^+0.7^	98.6^+0.2^	76.7^+4.7^	95.2^+0.9^	0.182
Model E	MG-Conv	HGSAM	FHP	85.3^+1.2^	**99.0^+0.6^**	**76.9^+4.9^**	95.8^+1.5^	0.190
Model F	MG-Conv	HGSAM	PCRH-P	85.3^+1.2^	**99.0^+0.6^**	**76.9^+4.9^**	**96.3^+2.0^**	0.192

## Data Availability

The data that support the findings of this study are openly available at https://share.phys.ethz.ch/~gseg/pairwise_reg/3dmatch.zip (accessed on 17 June 2025) and https://www.cvlibs.net/datasets/kitti/eval_odometry.php (accessed on 16 December 2025), and additional data related to this study are available upon reasonable request to the corresponding author.

## Abbreviations

The following abbreviations are used in this manuscript:

MGCE	Multivariate Geometric Combination Encoding
HGSAM	Hybrid Geometric-State Aggregation Module
PCRH-P	Physically Consistent Robust Hypothesis Proposer
VN	Vector Neuron
PPF	Point Pair Feature
SSM	State Space Model
RANSAC	Random Sample Consensus
Local-GSA	Local Geometric Self-Attention
PIR	Patch Inlier Ratio
IR	Inlier Ratio
RR	Registration Recall
FMR	Feature Matching Recall
RTE	Relative Translation Error
RRE	Relative Rotation Error
RMSE	Root Mean Square Error
